# A Label-Free Electronic Biosensor for Detection of Bone Turnover Markers

**DOI:** 10.3390/s91007957

**Published:** 2009-10-12

**Authors:** Yeo-Heung Yun, Amit Bhattacharya, Nelson B. Watts, Mark J. Schulz

**Affiliations:** 1 Nanoworld and Smart Materials and Devices Laboratory, College of Engineering, University of Cincinnati, Cincinnati, OH 45221, USA; E-Mail: mark.j.schulz@uc.edu; 2 Environmental Health, College of Medicine, University of Cincinnati, Cincinnati, OH 45221, USA; E-Mail: bhattaat@ucmail.uc.edu; 3 College of Medicine, University of Cincinnati Bone Health and Osteoporosis Center, 222, Piedmont Avenue, Suite 6300, Cincinnati, OH 45219; E-Mail: nelson.watts@uc.edu

**Keywords:** immunosensor, label-free, electrochemical impedance, bone turnover marker

## Abstract

This paper describes the development of a biosensor based on label-free immunosensing for the detection of the *C*-terminal telopeptide bone turnover marker from type-1 collagen. A self-assembled monolayer (SAM) of dithiodipropionic acid was deposited on a gold electrode. Then streptavidin and biotinylated anti-human C-terminal telopeptide antibody were successively conjugated on the self-assembled monolayer. Electrochemical impedance measurements were made to characterize each step of the SAM/streptavidin/biotinylated antibody binding. Subsequently, electrochemical impedance was measured with different concentrations of *C*-teminal telopeptide. A detection limit of 50 ng/mL and a dynamic range up to 3 μg/mL were achieved. To our knowledge, this is the first attempt to develop a label-free immunosensor based on electrochemical impedance with DC bias for detection of bone-related degradation and rebuilding products. The electronic biosensor might eventually be used for quantitative point-of-care screening of bone health. It is hoped that analysis of bone turnover markers can indicate the beginning of bone diseases such as osteoarthritis and osteoporosis so that treatment might start early when it is most effective.

## 1. Introduction

As a living tissue, the health of bone is characterized by how well the cellular mechanism adapts the bone to external loading through the modeling and remodeling processes. Both modeling and remodeling processes modify the size, shape, and internal micro and macro architecture of bone thereby directly influencing bone's mechanical structural integrity and its strength [1,2].

Bone turnover markers are useful in understanding bone physiology and assessing the action of medications [3,4]. Bone markers can also be used to assess fracture risk and to determine the response of bone to treatment. Bone turnover markers actually reflect the metabolic activity of bone during the modeling and remolding phases [5–7]. Biochemical bone markers reflecting remodeling include resorption markers such as collagen cross-links, while alkaline phosphatase is an example of a marker of bone formation [3,8]. Among several bone turnover markers reported in the literature [9,10], collagen is the main extracellular protein in the body and forms more than 25% of bone by volume. Collagen also constitutes 65% of the total organic component of bone tissue and is thus a good marker to study bone health [5]. During the remodeling of bone matrix throughout the skeleton, type I collagen is degraded and small peptide fragments are secreted into the blood stream [11]. In addition, pyridinium cross-link, cross-linked *N*-terminal telopeptides of type I collagen, and *C*-terminal telopeptides of type I collagen are released into urine [6]. Antibody is commercially available that is specific for the β-isomerized amino acid sequence (EKAHD-β-GGR) found in the *C*-terminal telopeptides of the α1 chain of type I collagen. Based on a previous electronic biosensor [12], a label-free electrochemical immunosensor for the detection of bone-related degradation products of *C*-terminal telopeptides from Type-1 collagen was developed and reported in this paper.

Bone turnover markers are currently under study as a clinical research tool, but they are not measured frequently in the same patient. Part of the problem is that they vary across the day and from day to day, so some way of integrating the levels might improve their utility. Presently, most methods to detect bone turnover markers are based on the enzyme-linked immunosorbent assay (ELISA) [11]. This method is time consuming, expensive, and consists of several steps including a staining procedure with secondary antibody and fluorescence measurement. In order to carry out a comprehensive study of patients to identify the metabolism of bone turnover [12–19], it would be useful to have an electrochemical biosensor as a point-of-care device that can quantitate several bone turnover markers at a relatively low cost. The objective of this paper is therefore to develop an electronic label-free biosensor based on electrochemical impedance spectroscopy (EIS) for the detection of bone turnover markers. The advantage of EIS sensing is label-free operation which does not need any secondary labeling method. EIS can be also used for multiple protein detection in real time. Besides specific proteins [12,14,15], DNA sequences [13] can also be detected using EIS. Even live-cell activity such as adhesion and proliferation was measured using EIS [16,17]. Recently aptamer [20] and peptide nucleic acids (PNA) [21] were immobilized on an electrode surface for the detection of a specific protein, or real time multiple markers [22–24] which increased sensitivity and lowered the detection limit. The EIS sensing approach described herein is generic and can be applied to many different bone markers by using different antibodies.

The electronic biosensor might provide additional information about bone turnover markers such as marker sensitivity and specificity useful for monitoring patients with certain bone diseases such as osteoporosis [30–34]. For example, bone turnover markers can be used monitor treatment [26–30] and improve patient persistence with therapy [31]. In addition, these markers are independent predictors of fracture risk [26] even though bone mineral density (BMD) assessment is still a major way [25].

## 2. Experimental Setup

### 2.1. Reagents and Materials

Biotinylated antibody and antigen (Serum CrossLaps ELISA kit) were purchased from Nordic Bioscience Diagnostics. Streptavidin was purchased from BD Pharmingen. All chemical reagents including K_3_Fe(CN)_6_, K_4_Fe(CN)_6_, and KNO_3_ were purchased from Sigma-Aldrich and prepared fresh daily in deionized water. Two different lengths of thiols and sulfides, 3,3-dithiodipropionic acid and 16-mercaptoundecanoic acid bearing carboxylic acid terminals were obtained from Sigma Aldrich. 1-Ethyl-3-[3-dimethylaminopropyl] carbodiimide hydrochloride (EDC) and *N*-hydroxy-sulfosuccinimide (Sulfo-NHS) were purchased from Pierce Biotechnology.

### 2.2. Instrumentation

In order to evaluate the electrochemical properties of electrodes, cyclic voltammetry (CV) and electrochemical impedance spectroscopy (EIS) were performed using three-electrode cells, with a gold electrode as the working electrode, an Ag/AgCl reference electrode, and a platinum wire counter electrode. CV was carried out using a Bioanalytical systems (BAS) analyzer operated with the Epsilon system including a C3 cell stand in a Faraday cage. EIS measurements were performed using a Gamry Potentiostat (Model: PCI4/750) coupled with EIS (Gamry, EIS300) software.

### 2.3. Gold Electrode Preparation and Antibody Immobilization

Gold electrodes were prepared using a routine photolithography method. First, hexamethyldisilazane (HMDS) was spin-coated on Si/SiO_2_ wafers. Then photoresist (S1818, Shipley Company, MA) was spin-coated on the wafer and soft-baked for 60 seconds at 115 °C. A shadow mask was used to selectively irradiate the photoresist with UV light for transferring the electrode design. A solitec mask aligner was used to align the optical path and expose the photoresist for 7 seconds. The wafers were then placed in developing solution and rinsed in deionized (DI) water. Gold (0.5 micron) was evaporated onto the patterned substrates using a Temescal FC1800 E-Beam Evaporator. A lift-off procedure was performed to remove the photoresist by soaking in acetone. SU-8 photoresist was spun on the pattern and soft-baked for use as an electrical insulator. Conducting epoxy was used to connect electrical wires to the gold electrode. SU-8 photolithography was used to insulate the whole area except the 1 mm diameter gold circle and square electrode. The final electrode was treated with oxygen plasma to clean the surface.

**Figure 1.**
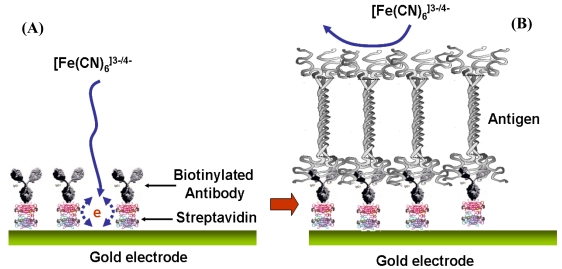
Schematic representation of a label-free immunosensor for bone maker detection. Part (A) shows a self-assembled monolayer of dithiodipropionic acid deposited on a gold surface with streptavidin immobilized next as a self-assembled monolayer. Then the biotinylated antibody was bound to the streptavidin. Part (B) illustrates the antigen-antibody binding event and how it hinders the interfacial electron transfer reaction of [Fe(CN)_6_]^3−/4−^.

The gold electrodes were again cleaned with piranha solution for 30 seconds and rinsed with ethanol successively and dried with a stream of nitrogen. The cleaned electrodes were placed in ethanol solution with 5 mM of dithiodipropionic acid and 16-mercaptoundecanoic acid overnight to form a self-assembled monolayer (SAM). Then electrodes were rinsed with ethanol in order to remove non-bonded thiols. The thiol functionalized electrodes were immersed in 0.5 mM of EDC and 0.1 mM of sulfo-NHS in pH 6.0 MES buffer solution successively to convert the terminal carboxylic groups to an active NHS ester. After rinsing the electrodes with DI water, the activated electrodes were immersed in 10 μg/mL of streptavidin in 10 mM of 7.4 pH, Phosphate Buffer Saline (PBS). After washing the electrodes to remove excess streptavidin, the electrodes were dipped in 10 μg/mL of biotinylated antibody in PBS solution for 4 hours. Then, after rinsing the antibody-modified electrodes in PBS, the antibody-electrodes were blocked using 1% bovine serum albumin (BSA) for 30 min to minimize non-specific binding sites. After washing the antibody modified electrodes in PBS, the electrodes were dipped into the different concentrations of antigen solution for electrochemical measurements. Immobilization of antibody and antigen was checked by using fluorescent labeled antibody. Another technique such as surface plasmon resonance (SPR) should be used in the future to enhance this check. Figure 1 is a schematic representation of the construction and operation of the label-free immunosensor for bone maker detection.

## 3. Results and Discussion

**Figure 2.**
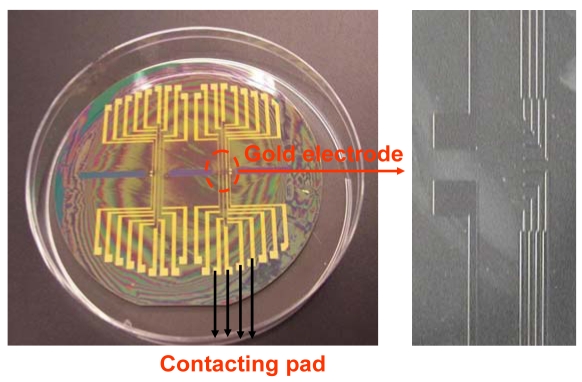
Optical picture of gold array electrodes which were patterned by a lithography technique.

**Figure 3.**
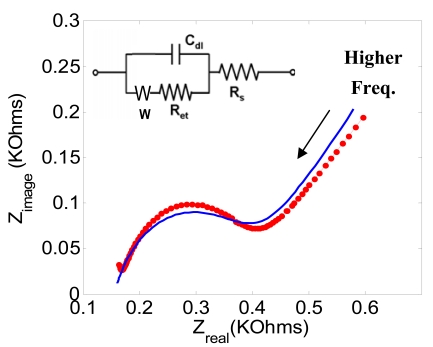
Electrochemical impedance spectra for a cleaned gold electrode at 0.2 V over a frequency range between 0.1 Hz and 300 kHz. The sinusoidal potential magnitude is 20 mV in 5.0 mM K_3_Fe(CN)_6_ and 5.0 mM of K_4_Fe(CN)_6_ in PBS (pH 7.0). The inset is an equivalent circuit model used to fit the experimental data. *C_dl_*, is the double layer capacitance; *R_et_*, is the electron transfer resistance; *W*, is Warburg impedance; and *R_s_*, is the solution resistance. The final curve-fit theoretical data in curve b matched well with curve a.

Figure 2 illustrates that different size of gold electrode arrays are fabricated using typical lithography techniques. Impedance measurement of a cleaned gold electrode was performed at 0.2 V over a frequency range between 0.1 Hz and 300 kHz. As shown in Figure 3(a), the overall EIS impedance is less than 1 KΩ and a well defined semi-circle was obtained. This experimental result was fit to Randle's equivalent circuit (Figure 3 Inset). The final curve-fit theoretical data in curve b matched well with curve a. These data are used as the baseline for further EIS study of the antibody-antigen binding complex.

**Figure 4.**
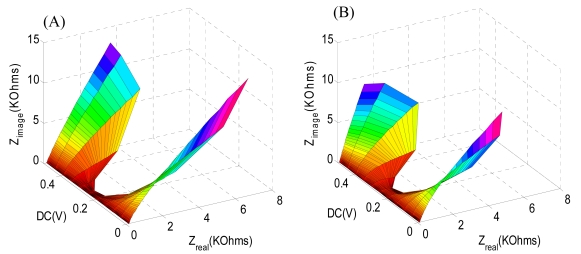
Three dimensional electrochemical impedance spectra of: (A) a self-assembled monolayer with carboxylic terminals on a gold electrode; and (B) a streptavidin immobilized electrode. EIS was done at DC potentials from 0 V to 0.5 V with frequencies between 0.1 Hz and 300 KHz. The sinusoidal potential magnitude was 20 mV in 5.0 mM K_3_Fe(CN)_6_ and 5.0 mM of K_4_Fe(CN)_6_ in PBS (pH 7.0).

Figure 4(A) shows the three dimensional EIS of the self-assembled monolayer of the carboxylic-terminated group on the gold surface in PBS with 5.0 mM K_3_Fe(CN)_6_ and 5.0 mM of K_4_Fe(CN)_6_. Impedance semi-circles occurred from 0.15 to 0.25 V DC potential, which represents the electron transfer resistance. In fact, the smallest semi-circle (lowest impedance) was found at 0.2 V DC for redox cycling of 5.0 mM K_3_Fe(CN)_6_ and 5.0 mM of K_4_Fe(CN)_6_. After immobilizing streptavidin on the carboxylic-terminated group on a gold electrode, the semi-circle diameter increased corresponding to higher electron transfer resistance as shown in Figure 4(B). The change in electron transfer resistance between Figure 4(A) and Figure 4(B) with the DC potential was calculated and the EIS at 0.2 V showed the largest change. Also, the three dimensional EIS was done more than 40 times and the change in electron transfer resistance between the self-assembled monolayer and streptavidin immobilized on the gold electrode was repeatable with less then 5% deviation for the 0.2 V DC potential. On the other hand, it was difficult to obtain repeatability above a 0.3 V DC potential. The 3-D plot provides general results for the whole range of voltage and shows how impedance changes with different DC potential. Thus, EIS was carried out at 0.2 V DC potential for the rest of the experiments.

**Figure 5.**
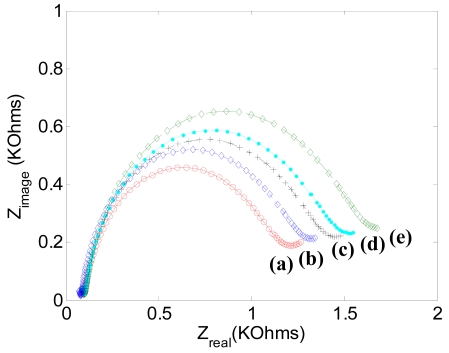
Electrochemical impedance spectra response recorded for the biotinylated anti-human C-terminal telopeptides antibody with the incubation time of one-half (a), one (b), two (c), four (d), and 24 hours (e). EIS was done at a DC potential of 0.2 V with frequencies between 0.1Hz and 300 KHz. The sinusoidal potential magnitude is ±20 mV in 5.0 mM K_3_Fe(CN)_6_ and 5.0 mM of K_4_Fe(CN)_6_ with PBS (pH 7.0).

Next, binding of the biotinylated anti-human *C*-terminal telopeptide antibody was done for different incubation times using a constant concentration, 10 μg/mL of antibody. In Figure 5, the influence of biotinylated antibody incubation time on EIS response shows that the electron transfer resistance gradually increases with the incubation time up to 4 hours. The increasing electron transfer resistance with time indicates that more biotinylated antibody is binding on the streptavidin on the gold electrode. Figure 5 shows that binding of the biotinylated antibody is dominant for the first two hours of incubation time. Thus the electrode was incubated with antibody for two hours for the subsequent antigen detection experiments. In this experiment the electrode became saturated with incubation time. Especially important is that there was no increase in circle diameter after 24 hours incubation. In this experiment, we did not stir the solution during antibody incubation which might be the reason the circle diameter increased gradually until a steady-state value was reached.

**Figure 6.**
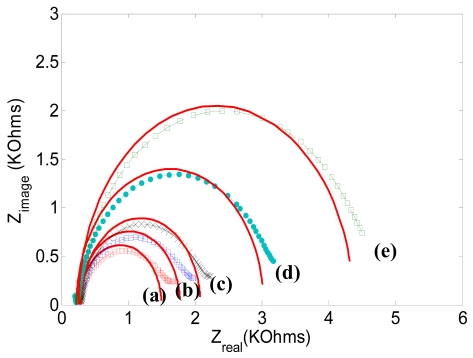
Electrochemical impedance spectra response recorded at the biotinylated anti-human C-terminal telopeptide antibody modified electrode in the presence of increasing concentration of human C-terminal telopeptide: 0 (a), 0.2 (b), 0.5 (c), 1(d), and 10 (e) μg/mL concentration of antigen. EIS was done at a DC potential of 0.2 V at frequencies between 0.1 Hz and 300 KHz. The sinusoidal potential magnitude was ±20 mV in 5.0 mM K_3_Fe(CN)_6_ and 5.0 mM of K_4_Fe(CN)_6_ in PBS (pH 7.0).

The procedure for the antigen detection experiment is to: (i) incubate the biotinylated antibody on the streptavidin functionalized electrode; (ii) apply antigen to the solution; (iii) incubate for 4 hours; (iv) wash the electrode with PBS to remove excess antigen; and (v) measure the EIS. This procedure was repeated for different concentrations of antigen. Figure 6 shows the EIS spectra of the immunosensor response recorded at the biotinylated antibody with the increase of C-terminal telopeptide (antigen) concentration. In Figure 6, the diameter of the semicircle, and hence the corresponding electron transfer resistance, was observed to increase with increasing concentration of antigen.

As shown in Figure 6, the electron transfer of the redox couple was impeded by antigen binding on the electrode surface. In such an experiment, a redox reaction with electron transfer kinetics as the rate-determining step will display a semicircular feature in an impedance plot of the imaginary component of impedance (*Z_image_*) versus the real component of impedance (*Z_real_*) over a frequency range. The diameter of the semicircle corresponds to the electron transfer resistance, *R_et._* Figure 6 shows the sequential impedance plots obtained after antigen binding occurred at the antibody modified electrode. In this Figure, the diameters of the respective semicircles were observed to increase, indicating an increasing electron transfer resistance for the [Fe(CN)_6_]^3−/4−^ redox reaction following the increase of antigen concentration. The EIS results can be simplified to a typical Randle's circuit model without the Warburg impedance term shown in the inset of Figure 3. Randle's circuit is an equivalent circuit representing each component at the interface and in the solution during an electrochemical reaction for comparison with the physical components; *C_dl_*, which is the double layer capacitor; *R_et_*, the electron transfer resistance; and *R_s_*, the solution resistance. Randle's circuit can be expressed as:

(1) $$Z(\omega )={\text{R}}_{et}+\frac{{\text{R}}_{\text{et}}}{1+\omega^{2}R_{\text{et}}^{2}C_{dl}^{2}}-\frac{j\omega R_{et}^{2}C_{dl}}{1+\omega^{2}R_{et}^{2}C_{dl}^{2}}=Z_{\text{real}}+jZ_{\text{imag}}$$

Based on the equivalent circuit model, simulated impedance plots were generated to fit the experimental results shown as red lines in Figure 6. Acceptable curve fits were obtained using the parameters shown in Table 1. Among the three parameters, *Rs* is a property of the electrolyte solution and the distance between the working and reference electrodes, and is not affected much by the interfacial kinetics on the electrode's surface. On the other hand, *Cdl* and *Ret* are interfacial properties of the electrode/electrolyte and are affected by an insulating layer or a coating on the surface of the electrode. As shown in Table 1, there is a more significant change in *Ret* than in *Cdl*. Therefore, the electron transfer resistance is chosen as the parameter to quantify the immunosensor response due to different analyate concentrations.

**Figure 7.**
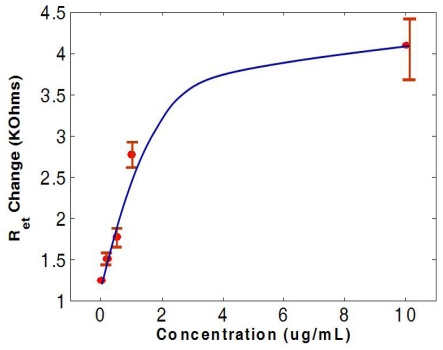
The relationship between the change in electron transfer resistance and the concentration of *C*-terminal telopeptide antigen.

Figure 7 shows the relationship between the change in electron transfer resistance and the concentration of antigen. The calibration curve shows a linear response up to 3 μg/mL and the results were repeatable within a 10% standard deviation.

**Figure 8.**
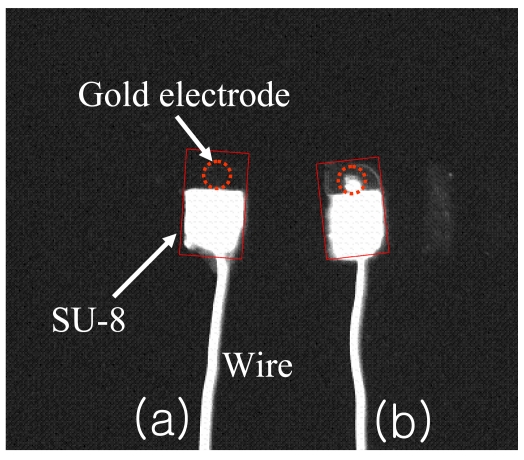
Fluorescence image of gold electrodes; (a) a gold electrode is coated with antibody and (b) a gold electrode is coated with antibody/antigen/fluorescence dye.

**Figure 9.**
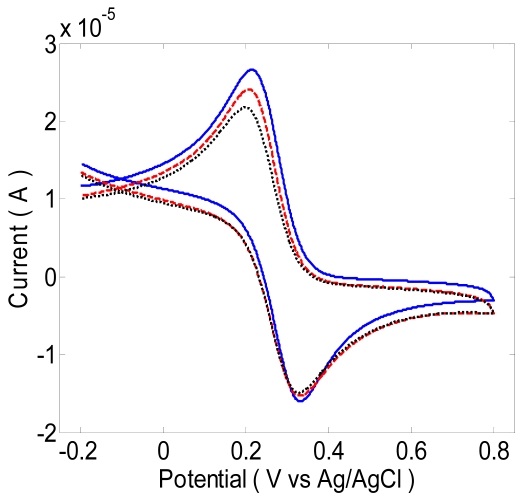
Cyclic voltammetry of 5.0 mM K_3_Fe(CN)_6_ and 5.0 mM of K_4_Fe(CN)_6_ in PBS (pH 7.0) with a 100 mV/s scan rate for: (a) the bare gold electrode; (b) the biotinylated anti-human *C*-terminal telopeptide antibody immobilized on the electrode after functionalizing with 3,3-dithiodipropionic acid; and (c) with 10 μg/mL human *C*-terminal telopeptide antigen in the solution.

Fluorescence microscopy using secondary antibodies was used to visualize the antigen-binding on the antibody-immobilized surface. As shown in Figure 8, there was no fluorescence on the antibody-coated electrode; however, the antigen incubated electrode showed bright fluorescence on the circle, and there was also auto-fluorescence on the SU-8 polymer. Figure 9 shows the cyclic voltammetry of 5.0 mM K_3_Fe(CN)_6_ and 5.0 mM of K_4_Fe(CN)_6_ in PBS (pH 7.0) with a 100 mV/s scan rate. With the immobilization of biotinylated anti-human *C*-terminal telopeptide antibody and *C*-terminal telopeptide antigen, the current slightly decreased. Blocking the electron transfer by antibody and antigen probably causes the decreasing current.

Repeatable results were obtained by normalizing the response of the sensor by the response of the sensor before binding. A detection limit of 50 ng/mL and a dynamic range up to 3 μg/mL range were obtained. Peichl *et al.* reported that *C*-terminal telopeptide concentrations are from 400 to 20,000 pmol/L in serum and from 30 to 1,400 μg/ mmol creatinine in urine. Thus, the suggested impedance sensor can cover the physiological levels of collagen *C*-terminal telopeptide. This type of immunosensor might be useful for rapid detection of bone markers. Optimizing the electrode size and improving the preparation of the surface of the electrode is expected to lower the detection limit dramatically. For future work, we are considering studying the sensitivity of the sensor in the presence of interfering materials such as EDTA, ascorbic acid, and other proteins that may cause non-specific binding. Simultaneous recording of multiple bone turnover markers at frequent time intervals would be possible with a multi-electrode electronic biosensor and this has not been practical in the past.

Other potentiodynamic methods are also good candidates for bone marker sensors. However amperometric detection often needs a secondary label and interference is another problem. Electrochemical impedance spectroscopy (EIS) is the complex measure of resistance, capacitance, and diffusion over a frequency range from 0.1 Hz to 100KHz. EIS usually provides more information and better sensitivity than individual component measurement or low frequency measurement techniques. Comparison of EIS with other potentiodynamic methods such as pulsed amperometric detection could be the subject of another paper.

## 4. Conclusions

We demonstrated a label-free immunosensor for the detection of bone-related degradation products of *C*-terminal telopeptides of type-1 collagen based on an electrochemical impedance method. There is a potential to increase the sensitivity by improving the preparation of the surface of the electrode and optimization of the electrode size. To our knowledge, this is the first attempt to develop a label-free immunosensor for the detection of bone-related degradation products, which might provide a quantitative point-of-care model to screen bone health and identify individual patients early who may be prone to osteoporosis. Compared to the ELISA commercial method, which has additional steps including secondary antibody immobilization with fluorescence dyes and further complex optical measurement, the suggested electronic sensor needs only one step (incubation) from the point of view of customers since the commercial biosensor would contain antibody-modified electrodes. Thus doctors or even patients can add the sample to the electrode as a point-of-care device and measure the electrical signal in 4 hours. Moreover, the electronic sensor can measure the concentration over a period of time whereas a single ELISA test cannot.

New information provided by the proposed biosensor may help to understand and predict bone disease. A measurement or “profile” for a patient might be done every 3–6 months at first and eventually less often. Clinical trials monitoring multiple bone markers simultaneously would help to understand the significance of changes in the bone markers over time and to establish a realistic measurement profile.

## Figures and Tables

**Table 1.** Estimated parameters for the equivalent circuit model of the immunosensor.

	**R_et_** [**KΩ**]	**C_dl_** [**μ*F***]	**R_s_** [**Ω**]
Figure 3 (a)	0.23	0.415	170
Figure 6 (a)	1.25	0.443	172
Figure 6 (b)	1.51	0.441	173
Figure 6 (c)	1.78	0.434	173
Figure 6 (d)	2.78	0.445	174
Figure 6 (e)	4.10	0.452	173

## References

[1] Seeman E., Delmas P.D. (2006). Bone quality-the material and structural basis of bone strength and fragility. N. Engl. J. Med..

[2] Ryouji M., Itsuo Y., Masahiko T., Yasuyo H., Itsuaki Y., Rikushi M. (2000). Comparison of various biochemical measurements with bone mineral densitometry and quantitative ultrasound for the assessment of vertebral fracture. J. Bone Miner. Metab..

[3] Watts N.B. (1999). Clinical utility of biochemical markers of bone remodeling. Clin. Chem..

[4] (2008). Review and search engine for osteoporosis. http://www.ucosteoporosis.com/.

[5] Burgeson R.E. (1998). Serum cross Laps one step ELISA: first application of monoclonal antibodies for measurement in serum of bone-related degradation products from C-terminal telopeptides of type I collagen. Annu. Rev. Cell. Biol..

[6] Rosenquist C., Fledeliu C., Christgau S., Pedersen B.J., Bonde M., Qvist P., Christiansen C. (1998). First application of monoclonal antibodies for measurement in serum of bone-related degradation products from C-terminal telopdptides of type I collagen. Clin. Chem..

[7] Okuno S., Inaba M., Kitatani K., Ishimura E., Yamakawa T., Nishizawa Y. (2005). Serum levels of C-terminal telopeptide of type I collagen: a useful new marker of cortical bone loss in hemodialysis patient. Osteoporosis Int..

[8] Ross P.D. (1999). Predicting bone loss and fracture risk with biochemical markers: A review. J. Clin. Dent..

[9] Wittich A., Casco C., Oviedo A., Zeni S., Nadal M., Mautalen C. (2001). Serum determination of C-terminal telopeptide of type 1 collagen (CTx) is a sensitive bone resorption marker in renal osteodystrophy. Bone.

[10] Hannon R.A., Eastell R. (2006). Bone markers and current laboratory assays. Cancer Treat. Rev..

[11] Srivastava A.K., Macfarlane G., Srivastava V.P., Mohan S., Baylink D.J. (2001). A new monoclonal antibody ELISA for detection and characterization of C-telopeptide fragments of type I collagen in urine. Calcified Tissue Int..

[12] Yun Y.H., Bange A., Heineman W.R., Halsall H.B., Shanov V.N., Dong Z., Pixley S., Behbehani M. (2007). A nanotube array immunosensor for direct electrochemical detection of antigen-antibody binding. Sens. Actuat. B.

[13] Ma K.S., Zhou H., Zoval J., Madou M. (2006). DNA hybridization detection by label free versus impedance amplifying label with impedance spectroscopy. Sens. Actuat. B.

[14] Rodriguez M.C., Kawde A.N., Wang J. (2005). Aptamer biosensor for label-free impedance spectroscopy detection of proteins based on recognition-induced switching of the surface charge. Chem. Commun..

[15] Belle J.T., Bhavsar K., Fairchild A., Das A., Sweeney J., Alford T.L., Wang J., Bhavanandan V.P., Joshi L. (2007). A cytokine immunosensor for multiple sclerosis detection based upon label-free electrochemical impedance spectroscopy. Biosens. Bioelectron..

[16] Ding L., Du D., Wu J., Ju H. (2007). A disposable impedance sensor for electrochemical study and monitoring of adhesion and proliferation of K562 leukaemia cells. Electrochem. Commun..

[17] Campbell C.E., Laane M.M., Haugarvoll E., Giaever I. (2007). Monitoring viral-induced cell death using electric cell–substrate impedance sensing. Biosens. Bioelectron..

[18] Peichl P., Griesmacher A., Marteau R., Hejc S., Kumpan W., Muller M., Broll H. (2001). Serum crosslaps in comparison to serum osteocalcin and urinary bone resorption markers. Clin. Biochem..

[19] Terpos E., Politou M., Rahemtulla A. (2005). The role of markers of bone remodeling in multiple myeloma. Blood Rev..

[20] Huang Y., Nie X.M., Gan S.L., Jiang J.H., Shen G.L., Yu R.Q. (2008). Electrochemical immunosensor of platelet-derived growth factor with aptamer-primed polymerase amplification. Anal. Biochem..

[21] Keighley S.D., Estrela P., Li P., Migliorato P. (2008). Optimization of label-free DNA detection with electrochemical impedance spectroscopy using PNA probes. Biosens. Bioelectron..

[22] Zhang Y., Wang H., Nie J., Zhang Y., Shen G., Yu R. (2009). Individually addressable microelectrode arrays fabricated with gold-coated pencil graphite particles for multiplexed and high sensitive impedance immunoassays. Biosens. Bioelectron..

[23] Levine P.M., Gong P., Levicky R., Shepard K.L. (2009). Real-time, multiplexed electrochemical DNA detection using an active complementary metal-oxide-semiconductor biosensor array with integrated sensor electronics. Biosens. Bioelectron..

[24] Deng C., Chen J., Nie Z., Wang M., Chu X., Chen X., Xiao X., Lei C., Yao S. (2009). Impedimetric aptasensor with femtomolar sensitivity based on the enlargement of surface-charged gold nanoparticles. Anal. Chem..

[25] Jackson B.F., Dyson P.K., Lonnell C., Verheyen K.L., Pfeiffer D.U., Price J.S. (2009). Bone biomarkers and risk of fracture in two- and three-year-old Thoroughbreds. Equine Vet. J..

[26] Garnero P., Hausherr E., Chapuy M.C., Marcelli C., Grandjean H., Muller C., Cormier C., Bréart G., Meunier P.J., Delmas P.D. (1996). Markers of bone resorption predict hip fracture in elderly women: The EPIDOS prospective study. J. Bone Miner. Res..

[27] Watts N.B., Jenkins D.K., Visor J.M., Casal D.C., Geusens P. (2001). Comparison of bone and total alkaline phosphatase and bone mineral density in postmenopausal osteoporotic women treated with alendronate. Osteoporos Int..

[28] Greenspan S.L., Parker R.A., Ferguson L., Rosen H.N., Maitland-Ramsey L., Karpf D.B. (1998). Early changes in biochemical markers of bone turnover predict the long-term response to alendronate therapy in representative elderly women: a randomized clinical trial. J. Bone Miner. Res..

[29] Eastell R., Barton I., Hannon R.A., Chines A., Garnero P., Delmas P.D. (2003). Relationship of early changes in bone resorption to the reduction in fracture risk with risedronate. J. Bone Miner. Res..

[30] Delmas P.D., Vrijens B., Eastell R., Roux C., Pols H.A., Ringe J.D., Grauer A., Cahall D., Watts N.B. (2007). Effect of monitoring bone turnover markers on persistence with risedronate treatment of postmenopausal osteoporosis. J. Clin. Endocrinol. Metab..

[31] Looker A.C., Bauer D.C., Chesnut C.H., Gundberg C.M., Hochberg M.C., Klee G., Kleerekoper M., Watts N.B., Bell N.H. (2000). Clinical use of biochemical markers of bone remodeling: current status and future directions. Osteoporos Int..

[32] Watts N.B. (1999). Clinical utility of biochemical markers of bone remodeling. Clin. Chem..

[33] Kleerekoper M., Camacho P. (2005). Monitoring osteoporosis therapy. Clin. Chem..

[34] Camacho P.M., Lopez N.A. (2008). Use of biochamical markers of bone turnover in the management of postmenopausal osteoporosis. Clin. Chem. Lab. Med..

